# Reply to: “Urine Dipstick Proteinuria and Cholelithiasis”

**DOI:** 10.2188/jea.JE20210354

**Published:** 2021-11-05

**Authors:** Sung Keun Park, Jae-Hong Ryoo

**Affiliations:** 1 Total Healthcare Center, Kangbuk Samsung Hospital, Sungkyunkwan University, School of Medicine, Seoul, Korea; 2 Departments of Occupational and Environmental Medicine, School of Medicine, Kyung Hee University, Seoul, Korea

To the editors,

We appreciate Shih-Wei Lai for his interest and valuable comments on our study.^1^ In the present study, we analyzed the risk of cholelithiasis according to the degree of urine dipstick proteinuria.^2^ Our analysis showed that heavy proteinuria group (urine dipstick proteinuria ≥2+) had the higher risk for incident gallstone compared with the negative proteinuria group. This finding suggests that urine dipstick proteinuria ≥2+ is potentially associated with the increased risk factor for cholelithiasis.

Nonetheless, we acknowledge that Lai’s points are reasonable and proper. Studies have presented that urine dipstick test is limited in accurately quantifying proteinuria.^3^^,^^4^ Urine dipstick proteinuria can be affected by physiological conditions, like dehydration and orthostatic position, other than pathologic problems.^5^ However, it is recognized that our study participants with increased urine dipstick proteinuria tended to have worse renal function. In our study groups, urine dipstick proteinuria showed an inverse relationship with estimated glomerular filtration rate (eGFR) and a proportional relationship with serum creatinine. Additionally, compared with negative urine dipstick proteinuria, the increased risk of cholelithiasis was observed only in the heavy proteinuria group (urine dipstick proteinuria ≥2+) presenting worst renal function. These findings imply that poor renal function may mediate the association between urine dipstick proteinuria and the risk of cholelithiasis. Therefore, The Chronic Kidney Disease Epidemiology Collaboration (CKD-EPI) equation was used to calculate eGFR.^6^ Our analysis indicated that the risk of cholelithiasis was inversely associated with quartile levels of eGFR (Table 1). Compared with the first quartile group of eGFR, adjusted hazard ratios (HRs) and 95% confidence intervals (CIs) for cholelithiasis significantly decreased with the increase of quartile levels of eGFR (quartile 1: reference, quartile 2: HR 0.85; 95% CI, 0.76–0.94, quartile 3: HR 0.87; 95% CI, 0.78–0.97, and quartile 4: HR 0.75; 95% CI, 0.67–0.83). These findings indicate that poor renal function is potentially associated with the development of cholelithiasis. Considering heavy urine dipstick proteinuria and poor renal function are associated with the increased risk of cholelithiasis, urine dipstick proteinuria in our study participants may be a marker for renal pathology rather than physiological response. Thus, it is postulated that increased urine dipstick proteinuria indicates the presence of renal disease, leading to the increased risk of cholelithiasis.

**Table 1.** Hazard ratios (HRs) and 95% confidence intervals (CI) for the incidence of cholelithiasis according to the quartile levels of eGFR

	Person-years	Incidence cases	Incidence rate	HRs (95% CI)^*^	

				unadjusted	multivariate adjusted model
**eGFR**					
Quartile 1	224,898.5	840	37.3	1.00 (reference)	1.00 (reference)
Quartile 2	226,101.3	717	31.7	0.85 (0.77–0.94)	0.85 (0.76–0.94)
Quartile 3	229,639.7	741	32.2	0.86 (0.78–0.95)	0.87 (0.78–0.97)
Quartile 4	223,721.0	621	27.7	0.74 (0.67–0.83)	0.75 (0.67–0.83)
*P* for trend				<0.001	<0.001

CI, confidence interval; eGFR, estimated glomerular filtration rate (mL/min/1.73 m^2^); HR, hazard ratio.

Multivariate adjusted model was adjusted for gender, body mass index, systolic blood pressure, fasting glucose, total cholesterol, gamma-glutamyl transferase, smoking amount (pack-years), alcohol intake, and physical activity.

Quartile 1: eGFR <71.08, Quartile 2: eGFR 71.08–83.10, Quartile 3: eGFR: 83.11–95.06, Quartile 4: eGFR >95.06.

Nonetheless, we agree with Lai’s opinion that urine dipstick proteinuria is not an independent risk factor for cholelithiasis. Indeed, overall incidence of cholelithiasis was low (1.41%), and that in the heavy proteinuria group was not so high (2.39%). Our results only show that the group with heavy urine dipstick proteinuria had a higher risk of cholelithiasis than the group with negative urine dipstick proteinuria. However, these findings do not guarantee the reliability and cost-effectiveness of urine dipstick test for proteinuria in predicting cholelithiasis. Therefore, further studies should be conducted to identify the pathological mechanisms and mutual interactions among urine proteinuria, renal disease, and cholelithiasis. These studies may reveal the useful markers for cholelithiasis regarding renal pathology.
